# Phonon transport control by nanoarchitecture including epitaxial Ge nanodots for Si-based thermoelectric materials

**DOI:** 10.1038/srep14490

**Published:** 2015-10-05

**Authors:** Shuto Yamasaka, Yoshiaki Nakamura, Tomohiro Ueda, Shotaro Takeuchi, Akira Sakai

**Affiliations:** 1 Graduate School of Engineering Science, Osaka University, Toyonaka, Osaka 560-8531, Japan; 2 PRESTO, JST, 4-1-8 Honcho Kawaguchi, Saitama 332-0012, Japan

## Abstract

Phonon transport in Si films was controlled using epitaxially-grown ultrasmall Ge nanodots (NDs) with ultrahigh density for the purpose of developing Si-based thermoelectric materials. The Si/Ge ND stacked structures, which were formed by the ultrathin SiO_2_ film technique, exhibited lower thermal conductivities than those of the conventional nanostructured SiGe bulk alloys, despite the stacked structures having a smaller Ge fraction. This came from the large thermal resistance caused by phonon scattering at the Si/Ge ND interfaces. The phonon scattering can be controlled by the Ge ND structure, which was independent of Si layer structure for carrier transport. These results demonstrate the effectiveness of ultrasmall epitaxial Ge NDs as phonon scattering sources, opening up a route for the realisation of Si-based thermoelectric materials.

Thermoelectric (TE) materials, which convert wasted heat to electricity, have attracted considerable attention because they could provide a solution to energy problems^1^,^2^,^3^. The dimensionless figure of merit in TE conversion is defined as: *ZT *= *S*^2^*σT*/*κ*, where *S* is the Seebeck coefficient, *σ* is the electrical conductivity, *T* is the absolute temperature and *κ* is the thermal conductivity. This means that materials with low *κ*, high *S* and high *σ* values are desirable. Conventionally, in chalcogenide materials such as Bi_2_Te_3_ and PbTe, high *ZT* values have been achieved^4^,^5^. This is mainly because the heavy elements in materials lead to low *κ* values, while keeping relatively high *σ*. However, these materials include rare or toxic elements, which are not good for practical use. In contrast, ubiquitous materials, that is, eco-friendly and non-toxic materials have exhibited low *ZT* values. Recently, many studies have reported that material nanostructuring is quite effective for enhancing the TE performance^6^,^7^,^8^. Much attention is being paid to developing high *ZT* thermoelectric materials composed of ubiquitous elements such as Si-based materials using nanostructuring^9^,^10^,^11^,^12^,^13^,^14^,^15^,^16^,^17^,^18^. When Si is used as a TE material, its high power factor (*S*^2^*σ*) is desirable, but its high *κ* value is a large barrier for obtaining a high *ZT*. Therefore, reducing *κ* is essential.

In general, enhancing the TE performance by introducing nanostructures arises from phonons scattering at the interfaces related to the nanostructures, resulting in a reduction in *κ*. It was reported that nanostructures should be endotaxially or epitaxially introduced into materials to suppress the degradation of *σ*^19^. Recently, we reported nanostructured Si made of epitaxially- and coherently-connected Si nanocrystals with identical crystal orientations^20^. In the nanostructured Si, the oriented Si nanocrystals were separated by just a 1-monolayer (1-ML) thick SiO_2_ layer and connected only through nanowindows (<1 nm) in the SiO_2_ layer. The thermal resistance per interface was able to be increased by decreasing the size of the Si nanocrystals. This structure succeeded in lowering *κ* to the value of amorphous Si. On the other hand, the ultrasmall size of the Si nanocrystals also led to the excessive increase in the density of the interfaces between connected Si nanocrystals in an uncontrollable way, which could also degrade *σ*.

In order to independently control the phonon and carrier transports, we propose an improved nanoarchitecture (see Fig. 1a) where ultrasmall epitaxial nanodots (NDs) are introduced as phonon scattering sources in epitaxial Si layers related to carrier transport. In this structure, the size and shape of the NDs can be tuned for phonon scattering and the Si layers can be designed to have high carrier mobilities for carrier transport, independently. Ge is one of the promising candidates as a ND material because of its compatibility with conventional Si process technologies and recently we developed the formation technique of the new nanoarchitecture using the ultrathin SiO_2_ film technique^21^. In this nanoarchitecture, ultrasmall Ge NDs were epitaxially grown with identical crystal orientations, which were completely different from the conventional nanostructured SiGe bulk alloys made by sintering. In addition, the Ge NDs in these structures are expected to effectively reduce *κ* because of the ultrasmall (nanoscale) ND size compared with the case of Ge NDs/Si superlattices that use the well-established Stranski-Krastanov (SK) growth mode, which have a typical ND size of ~30–100 nm^22^,^23^. Furthermore, unlike SK NDs, the interfaces between the ultrasmall Ge NDs and Si can be well-controlled; intermixing at the interfaces is suppressed and there are no strains and misfit dislocations in the Ge NDs^21^,^24^,^25^. Therefore, this nanoarchitecture is useful to control the phonon and carrier transport properties related to enhancing the TE performance and to clarify its mechanism.

Here, based on the strategy shown in Fig. 1a, lowering *κ* is focused on, which is essential to obtain high *ZT* Si-based TE materials, as mentioned above. A large reduction in *κ* (~1.2 Wm^−1^K^−1^) at a small Ge fraction (amount ratio of Ge and Si) of ~10% is achieved using our proposed nanoarchitecture, where the *κ* value is lower than those of conventional SiGe alloys^11^,^26^ and nanostructured SiGe bulk alloys^11^. The low *κ* value is attributed to the large interfacial thermal resistance in our proposed nanoarchitecture. The interfacial thermal resistance, which is a key factor in TE nanostructures, is larger than that of SK Ge NDs/Si superlattices^27^,^28^. The result demonstrates the possibility of independently controlling carrier transport by designing a Si layer and the phonon transport by tuning the structure of the Ge NDs.

## Methods

Chemically cleaned non-doped Si(001) substrates were introduced into a molecular beam epitaxy chamber equipped with a reflection high energy electron diffraction (RHEED) with a 20 keV electron beam at a base pressure of ~3 × 10^−8 ^Pa. Clean Si surfaces were prepared by epitaxially growing ~100-nm thick Si buffer layers on Si(001) substrates at 500 °C after degassing at 500 °C for several hours. The clean Si surfaces were then oxidized at 500 °C for 10 min at an oxygen pressure of 2 × 10^−4^ Pa to form ultrathin (<1 nm) SiO_2_ films^29^,^30^,^31^. 7–66 monolayers (MLs) of Ge were deposited on the ultrathin SiO_2_ films at 500 °C to form epitaxial Ge NDs^24^,^32^,^33^. Si (40–376 ML) was deposited on the Ge NDs at 400 °C to form epitaxial Si layers. The Si layers were then oxidized to form ultrathin (<1 nm) SiO_2_ films at 450 °C. The structures of the ultrathin SiO_2_ films/Si layers/Ge NDs, formed using the above three processes were defined as a ‘one cycle structure’. The formation of one cycle structure was repeated eight times to fabricate epitaxial stacked Si layer/Ge NDs structures. The stacked structure of *x*-nm diameter Ge NDs/*y*-ML Si layers is referred to as the “*x*-nm NDs/*y*-ML Si sample”. This technique for forming Si/Ge ND stacked structure was reported in our previous paper^21^.

**Figure 1:**
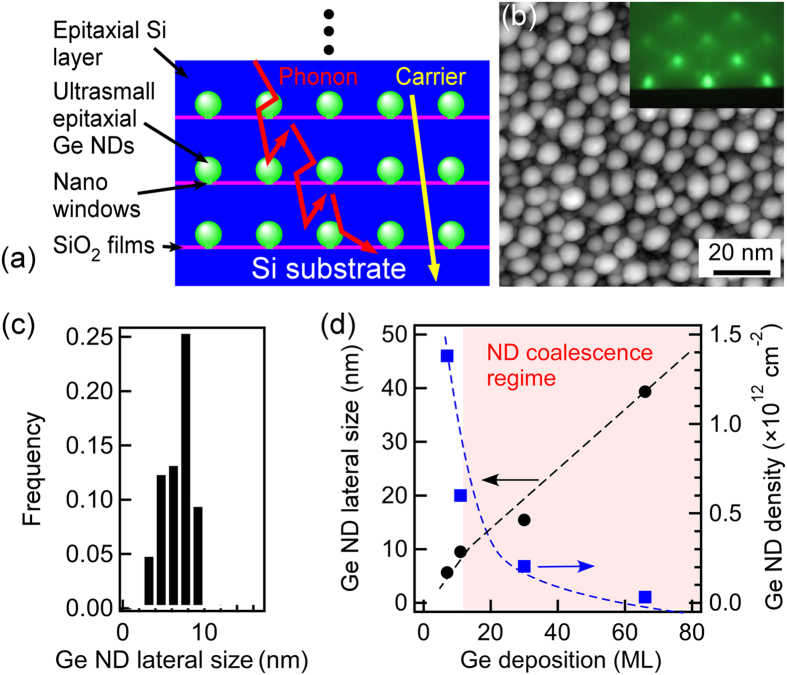
(**a**) Schematic of the proposed nanoarchitecture for a Si film containing Ge NDs as the phonon scattering sources. (**b**) STM image and RHEED pattern (inset) of Ge NDs on Si substrates formed by depositing 7 ML Ge on ultrathin SiO_2_ films. The incident electron beam was <110>_Si_ in direction and 20 keV in energy. (**c**) Size distribution of the Ge NDs, acquired from the STM images. (**d**) Dependence of the Ge ND size and density on the amount of Ge deposited. The dotted lines were inserted to guide the eyes.

Here, the formation mechanism of Ge NDs using ultrathin SiO_2_ films^24^ is briefly described. During the first stage of depositing Ge onto ultrathin SiO_2_ films, nanowindows with an ultrahigh density (>10^12^ cm^−2^) are fabricated on the ultrathin SiO_2_ films through the following reaction, Ge + SiO_2_ → GeO↑ + SiO↑. During subsequent Ge deposition, the nanowindows act as nucleation sites, leading to epitaxial growth of ultrasmall Ge NDs on the Si substrates. The density of Ge NDs is ultrahigh (~10^12^ cm^−2^) when the Ge ND size is small (<~10 nm). The ultrahigh density comes from the nanowindow separation of ~10 nm, which is determined by the diffusion length of the Ge atoms on the ultrathin SiO_2_ films^34^. Figure 1b and its inset show a scanning tunnelling microscopy (STM) image and a RHEED pattern with an electron beam incident direction of <110>_Si_ of the sample after depositing 7 ML Ge, respectively, as a typical example. This STM image shows the growth of Ge NDs with ultrahigh density (1.4 × 10^12^ cm^−2^) on Si substrate and RHEED pattern shows the epitaxial growth of these Ge NDs. The lateral size distribution of Ge NDs estimated from the STM image is shown in Fig. 1c, demonstrating an average lateral size of 5 nm and a sharp size distribution with a standard deviation of 1.7 nm.

The *κ* value was measured in the [001]_Si_ direction, perpendicular to the surface, using the 2ω method^20^,^35^,^36^ at room temperature (RT). In the 2ω method, 100-nm thick Au films were deposited on the surface of the stacked structures. Modulation heating was done by passing through the modulated electric current in the Au films. During the heating, a thermoreflectance signal related to the modulated temperature of Au film surfaces was measured by laser with wavelength of 635 nm. It was confirmed that the electric current only flew through the Au film without any current leaking into the underlying film and substrate by comparing the electrical resistances of Au films on the samples (a few Ω) and those of the samples without Au films (on the order of MΩ).

## Results and Discussion

**Figure 2:**
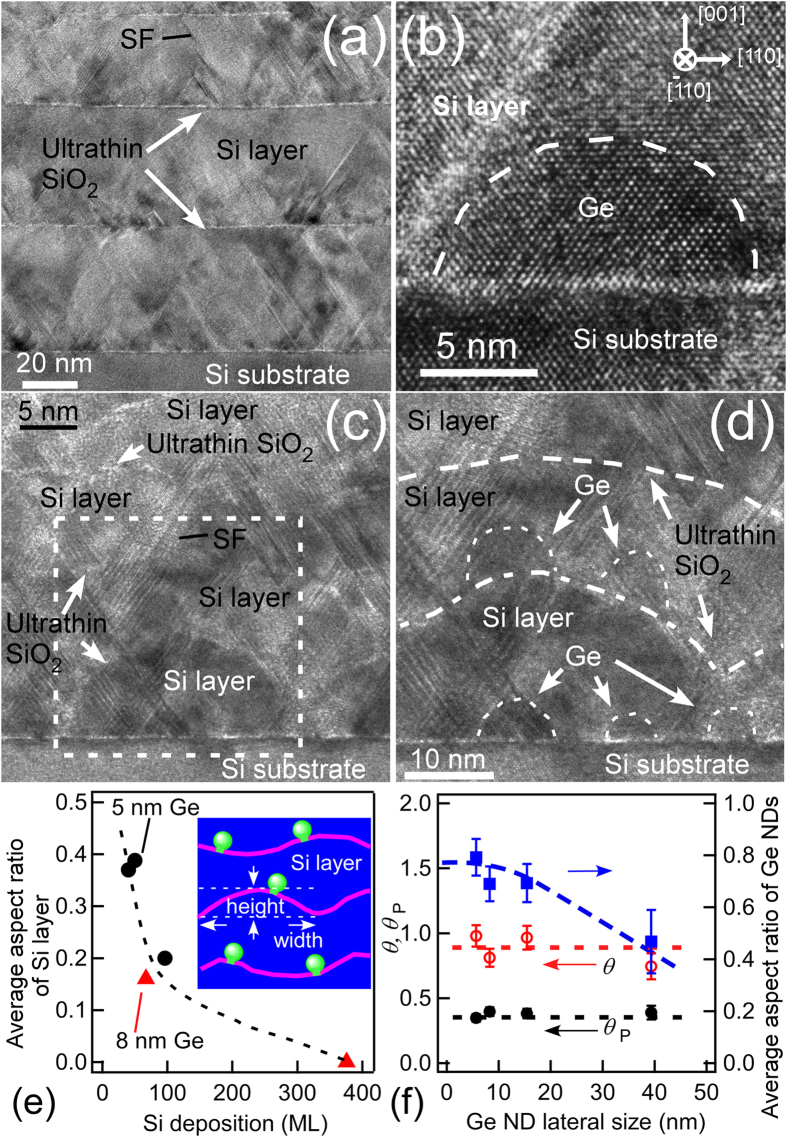
(**a**) Cross-sectional HRTEM image of an 8-nm NDs/376-ML Si sample. (**b**) Enlarged HRTEM image near the Si substrate of an 8-nm NDs/376-ML Si sample. (**c**) Cross-sectional HRTEM image of a 5-nm NDs/96-ML Si sample. (**d**) Enlarged HRTEM image of the dotted square region in (**c**). The crystal orientations of the HRTEM images are the same as the orientation described in the inset in (**b**). (**e**) The average aspect ratios of the Si layers (illustrated in the inset), were measured from the HRTEM images for the 5-nm NDs/*y*-ML Si and 8-nm NDs/*y*-ML Si samples. The dotted line was inserted to guide the eyes. (**f**) The interfacial area of the Si/Ge NDs per unit area on the (001)_Si_ plane, *θ* (open circles), the projection of *θ* on the (001)_Si_ plane*, θ*_P_ (solid circles), and the average aspect ratio of the Ge NDs (solid squares). The errors include the statistical errors in measurements and possible structure variation of Ge NDs formed on rough Si layers. The dotted lines were inserted to guide the eyes.

Figure 1d shows the dependences of the lateral size and the density of Ge NDs on the amount of Ge deposited on an ultrathin SiO_2_ film. When the ND lateral size was larger than ~10 nm, namely larger amount of Ge deposited (>~11 ML), the Ge ND lateral size increased monotonically with respect to the amount of Ge deposited. Simultaneously, the Ge ND density dropped from ~10^12 ^cm^−2^ for 7 ML Ge with an increase in the amount of Ge deposited, indicating that for a larger ND lateral size (>~10 nm), the Ge NDs grew and coalesced. This can be understood by considering the distance between nanowindows (~10 nm), which corresponds to the separation of the NDs with a constant density of ~10^12^ cm^−2^ in the small ND size range^20^. The average aspect ratios (height/lateral width) of the Ge NDs are shown in Fig. 2f.

Si layers/Ge NDs stacked structures were fabricated by the aforementioned process, during which epitaxial growth was confirmed by RHEED observations. Figure 2a shows a cross-sectional high-resolution transmission electron microscopy (HRTEM) image of an 8-nm NDs/376-ML Si sample, which is the case of thick Si layer. A stacked structure was observed, where the flat Si layers were separated periodically by ultrathin SiO_2_ films, as indicated by the arrows. Stacking faults (SFs), often forming twin boundaries were also observed in the stacked structure. The enlarged image of the region near the SiO_2_ film in Fig. 2b reveals that hemispherical Ge NDs with a dark contrast existed in the Si layer, and the NDs were on the ultrathin SiO_2_ films. For the case of the thinner Si layer (96 ML), the Si layer became rough and was not flat, as shown in Fig. 2c. The rough Si layers were stacked and separated by ultrathin SiO_2_ films with bright contrasts indicated by the arrows. Figure 2d is an enlarged image corresponding to the region in the dotted square in Fig. 2c. It is observed that the rough Si layers included hemispherical Ge NDs, similarly to the case for the aforementioned thick and flat Si layer. The roughening of the Si layers was caused by their growth on the Ge NDs with nanoscale roughness. The Si layers in the first cycle structures were mountain-like, while the Si layers in the second and further cycle structures were relatively flat and not mountain-like, as shown in Fig. 2c,d. To control the shape of the Si layers, the interfaces of which can cause a large thermal resistance, several stacked structures were fabricated with various Si deposition amount in one cycle structure, *y* (*y* > 40 ML). The average aspect ratios of the Si layers were evaluated from the HRTEM images as a function of *y* (see the inset in Fig. 2e). Figure 2e shows that the rough Si layers gradually became flatter with an increase in the amount of Si deposited, demonstrating that it is possible to control the shape of the Si layer.

In the discussion on the thermal transport, the interfacial area of the Si/Ge NDs is also an important factor in addition to the interfaces between the Si layer-Si layer because the Si/Ge ND interfaces can be phonon scattering sources. In the estimation of interfacial area of the Si/Ge NDs, it was assumed that a ND with height (*h*) and radius (*r*) is half a spheroid. The surface area of half a spheroid is either 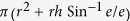 with 
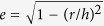
at *h* > *r*, or 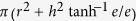 with 
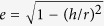
at *h* < *r*. The interfacial areas of the Si/Ge NDs per unit area on the (001)_Si_ plane (*θ*) can be estimated from the surface area of half a spheroid. The cross section of the NDs for the heat current is also an important factor for phonon transport, which corresponds to the projection of *θ* on the plane perpendicular to the heat current direction, that is the Si(001) plane (*θ*_p_). Although Ge NDs grew on rough Si layers in some cases, the structure factors of Ge NDs such as *h*, *r*, and projected widths of the ND lateral size on the Si(001) planes exhibited almost the same values in any *n*th cycle structure (*n *= 1–8) within the error, which were confirmed by analysis of HAADF-STEM (high-angle annular dark-field-scanning transmission microscopy) images reported in our previous work^21^ (see supplementary information). Therefore, the *θ* and *θ*_p_ in the first cycle structures were used as typical values in the sample, which were estimated from the STM images. Both *θ* and *θ*_p_ did not depend on the Ge ND lateral size within this size range, as shown in Fig. 2f.

**Figure 3:**
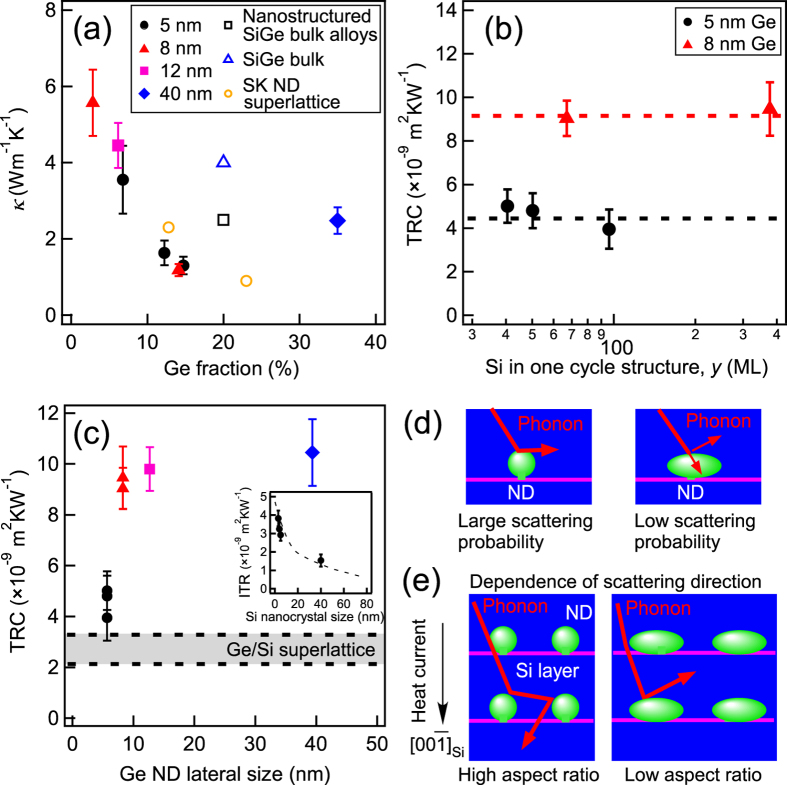
(**a**) Thermal conductivities (*κ*) of our Si/Ge ND stacked structures (Solid marks). Ge ND sizes in the stacked structures are described in the inset. For reference, those of conventional SiGe materials (nanostructured SiGe bulk alloy^11^: open squares, bulk SiGe^11^,^12^: open triangles), and superlattices of SK NDs^27^,^28^ (open circles) are plotted. (**b**) TRCs of 5-nm Ge NDs/*y*-ML Si and 8-nm Ge NDs/*y*-ML Si samples. (**c**) Dependence of the TRC on the Ge ND lateral size in our Si/Ge ND stacked structures. The band surrounded by dotted lines indicates ITR values of Ge/Si superlattices estimated by the theoretical calculation^41^,^42^ and experimental result (2.125 × 10^−9 ^m^2^KW^−1^)^27^. There are many calculated results: the finite-volume simulation of the phonon Bolzmann transport equation (3.3 × 10^−9^ m^2^KW^−1^)^41^, full dispersion diffuse mismatch model (2.72 × 10^−9^ m^2^KW^−1^)^41^, gray Bolzmann transport equation model (2.09×10^−9 ^m^2^KW^−1^)^41^ and atomistic Green’s functions (3.125 × 10^−9^ m^2^KW^−1^)^42^. The inset shows the ITR of Si nanocrystals^20^. (**d**) Schematics of phonons scattering from Ge NDs with different radii of curvature. (**e**) Schematics of the phonon transport through the stacked structures, including Ge NDs with different aspect ratios. The scattering direction is strongly dependent on the aspect ratio of the NDs.

The *κ* values of the Si/Ge NDs stacked structures were measured at RT, indicated by the solid marks in Fig. 3a. As a reference, the *κ* values of conventional bulk SiGe^11^,^12^, a nanostructured SiGe bulk alloy^11^ and a SK Ge NDs/Si superlattice^27^,^28^ are also indicated with open marks. An increase in the Ge fraction at the same Ge ND size corresponded to a decrease in the Si layer thickness. For the 8-nm NDs/67-ML Si sample, the *κ* value was reduced to ~1.2 Wm^−1^K^−1^. This demonstrated that these samples exhibited a lower thermal conductivity at a relatively small Ge fraction of ~10% than those of the other conventional SiGe materials at Ge fraction of 20%^11^,^12^. Nice theoretical paper predicted^18^ that in Si including Ge nanoinclusions which is similar to our structure, *κ* can be close to that of Si_0.5_Ge_0.5_ alloy (~5 Wm^−1^K^−1^). In our nanoarchitecture, the *κ* value got much smaller experimentally than this value, demonstrating the *κ* reduction beyond alloy limit. Furthermore, the *κ* value was lower than that of SK Ge NDs/Si superlattice at Ge fraction of ~10%^27^ and comparable to that of SK Ge NDs/Si superlattice at Ge fraction of ~20%^28^. This result shows that the proposed stacked structures reduce thermal conductivity the most effectively in the various SiGe materials studied. In general, the effect of reducing *κ* by nanostructuring is believed to be caused by the increase in the density of the interface with a relatively large thermal resistance. To remove the contribution of the stacked interfacial density from the discussion on the reduction of *κ*, the thermal resistance per cycle structure (TRC) was estimated by dividing the thermal resistance of the stacked structures by the stacking number. Figure 3b shows the TRC values for the 5- and 8-nm Ge NDs as a function of the Si layer thickness in one cycle structure, *y*. The constant TRC values for the same Ge ND size indicated that the TRC did not depend on the Si layer thickness or shape (Fig. 2e). This showed that the decrease of *κ* with an increase in the Ge fraction for the same Ge ND size (see Fig. 3a) came from the increase in the stacked interfacial density in the vertical direction related to the decrease in the Si layer thickness (namely the Ge fraction increase). Alternatively, the TRC strongly depended on the Ge ND size. This indicates that reducing *κ*, namely the large TRC, was mainly caused by the existence of Ge NDs. This implies that the thermal resistance was determined by the Ge ND size, independently of the morphology of the Si layer related to carrier transport. Furthermore, the TRC in our sample was larger than those of the SK ND superlattice, estimated from the reports by Pernot *et al.* (2–4 × 10^−9^ m^2^KW^−1^)^28^, indicating the effectiveness of the present Ge NDs.

The existence of SFs in the samples should also be considered as a mechanism that could cause the increase in the thermal resistance. However, the effect of the SFs is very small (~30% *κ* reduction in bulk fcc crystals)^37^ compared with the present reduction in *κ*. Therefore, SFs are not likely the main cause of the reduction in *κ*.

The insensitivity of TRC to the shape of the Si layer can easily be understood by considering our previous results^20^. According to the Si nanocrystal case^20^, which had a similar structure to the Si layers in the present case (Si layers covered with ultrathin SiO_2_ films), although the connected Si nanocrystals covered with ultrathin SiO_2_ films exhibited size-dependent interfacial thermal resistance (ITR) in the range of small nanocrystal width (<40 nm), which was monotonically decreasing, its thermal resistance was small (~1.5 × 10^−9^ m^2^KW^−1^) for the large nanocrystal case (~40 nm) (see the inset in Fig. 3c)^20^. This demonstrated that in the present case with a large Si layer (>~40 nm), the ITR of a Si layer had a smaller value than TRC of Si/Ge ND stacked structures, resulting in a small contribution of structural change of Si layer to TRC. To investigate the resultant large contribution of the Ge NDs to the increase in the TRC, the TRC is plotted as a function of Ge ND size in Fig. 3c. The TRC increased with an increase in the ND size and then saturated. Initially, it was thought that this might have resulted from variations in the interfacial area of the Ge NDs, behaving as phonon scattering sources. When a phonon cannot easily pass through the stacked structures and exists for a relatively long time, the phonon diffuses in Si films with Ge ND scattering sources. Then, the *θ* related to the ND interfacial area is an important factor for the phonon scattering. Conversely, when a phonon can easily pass through the stacked structures, the *θ*_p_ related to the cross section of the Ge NDs influences the phonon scattering. However, as shown in Fig. 2f, both *θ* and *θ*_p_ are almost constant in the Ge ND coalescence regime, and therefore in terms of the interfacial area of the Ge NDs, the size dependence of the TRC cannot be explained.

In general wave scattering theory, the wave scattering depends on the scatterer size. When the scatterer size is much smaller than the wavelength, Rayleigh scattering occurs, while the wave undergoes scattering analogous to Mie scattering in light wave when the scatterer size becomes larger than the wavelength. Considering the scattering in the whole range including the above-mentioned two scattering regimes, scattering efficiency increases with increase of the size parameter, *χ* defined by scatter diameter (=size) ×*π*/wavelength, and is saturated at *χ* of 6 ~ 10 in the case of isotropically-round scatterers^38^. This model can explain the ND size dependence of TRC in Fig. 3c qualitatively because TRC is directly related to scattering efficiency. However, in the Si case where the characteristic wavelength of phonons is reported to be ~1 nm^39^,^40^, the scattering efficiency should have been saturated at ND size of 2 ~ 3 nm. This indicates that the scattering efficiency in the ND size range of 5 ~ 40 nm is in the saturation region, which does not agree with the TRC result in Fig. 3c quantitatively. In practice, the phonon transporting heat has the width in the wavelength, and the ND scatterers have the anisotropic shape. Therefore, these factors should be considered in the wave scattering theory. Here, to investigate the scatterer shape effect roughly instead of the calculation of rigorous wave scattering including its effect, we discuss a simple scattering model as shown in Fig. 3e. Figure 3e shows that the phonons scattered by the round NDs had a more positive motion component along the heat current direction (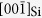) than the phonons scattered from the flat NDs, resulting in a relatively weak hindrance of the phonon transport along 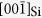. In this framework, with an increase in the Ge ND lateral size (>~10 nm), the average aspect ratio decreases (see Fig. 2f); the NDs became flattened, resulting in a larger TRC. This rough consideration demonstrates the scatterer shape effect is consistent with the tendency of Fig. 3c., indicating its tendency can also be explained by wave scattering theory including the shape effect.

In the above discussion, it was assumed that phonons are scattered with some probability at the hetero-interfaces. However, the origin of the scattering at the hetero-interface in the present case should also be considered in the above-discussed wave scattering. At the Si-Ge interfaces, Si-Ge mixing and the existence of strain should be considered for the phonon scattering. It has been reported that the Ge NDs formed using the present method are strain-relaxed and there is no mixing^21^ in our nanoarchitecture. Therefore, simple acoustic mismatch between Si and Ge has only to be discussed in consideration of the strain in the Si layers near the interfaces. However, novel phonon scattering at the interfaces with the nanoscale roughness was reported in the connected Si nanocrystal system^20^. When the curvature radius of the interface decreased to the nanoscale, the ITR increased, as shown in the inset in Fig. 3c. This tendency was explained by novel phonon scattering which was influenced by the curvature radius of interfaces as illustrated by Fig. 3d. Although the present Si/Ge ND interface is different from that of connected Si nanocrystals, it is believed that there is a similar tendency of the ITR on the curvature radius of the interface on the nanoscale^20^. However, this curvature radius dependence is opposite to that in Fig. 3c. Therefore, in the phonon transport/scattering in the present nanoarchitecture, there should be two competing effects to be considered: the curvature radius effect (Fig. 3d) and the shape effects (Fig. 3e) of ND interface which influence the phonon scattering probability and phonon scattering direction, respectively. The curvature radius effect is confirmed by comparing TRC of the Ge film/Si supperlattice and our nanoarchitecture. The Ge film/Si supperlattice structure, which is a flattening limit of Ge NDs (aspect ratio → 0), exhibited a smaller TRC than those in the present Ge ND case. TRC values of Ge films/Si supperlattices were acquired by many theoretical calculations^41^,^42^ and experimental study^27^, which are described by the band mark in Fig. 3c. The fact about large TRC in our nanoarchitecture than that of supperlattice is inconsistent with the wave scattering model with the ND shape effect, but can be explained by considering the curvature radius effect of ND interfaces as reported in our previous work^20^. The two competing effects complicate TRC dependence on ND size, but there can exist a proper Ge lateral size for prevention of phonon transport, which could be expected to be a few tens of nanometres in the Si/Ge ND structure from Fig. 3c.

Further theoretical study about phonon transport including the shape effect, curvature radius effects, or other effects is needed to clarify the mechanism for the large TRC value and the dependence of the TRC quantitatively because our proposed mechanism is just a qualitative model. However, in this study, the *κ* reduction effect resulting from large TRC in our proposed nanoarchitecture can give knowledge for thermoelectric nanomaterial study.

Although the present paper topic is *κ* reduction, we conducted a preliminary experiment about electrical measurement of this nanoarchitecture. As a representative, the electron mobility of 8-nm NDs/67-ML Si sample, which exhibited the lowest *κ* value (~1.2 Wm^−1^K^−1^), was measured to be ~370 cm^2^V^−1^s^−1^ at the carrier concentration of ~6.4 × 10^16^ cm^−3^ by Hall effect method and the Van der Pauw method, where the sample was doped by phosphorous ion implantation and activation annealing at 635 °C. This electron mobility was about 0.5 times as large as that of bulk Si at the same carrier concentration^43^. This reduction effect is small compared with *κ* reduction (1/120 times as large as that of bulk Si). Furthermore, the mean free path of electron decreases with increase of doping level in general. In the high doping level of ~10^19^–10^20^ cm^−3^ which is general doping range in practical use of TE materials, the electron mobility degradation from that of bulk Si should be suppressed because the mean free path of electron in Si was calculated to be a small value of ~10 nm^44^, which is smaller (comparable) than (to) the Si layer thickness in this nanoarchitecture. This implies that the Ge NDs and ultrathin SiO_2_ film were weak scattering sources for charge carriers in the stacked structures and the feasibility of independent control of electrical and thermal properties.

## Conclusions

In summary, an epitaxial stacked structure of ultrasmall Si/Ge NDs was fabricated using an ultrathin SiO_2_ film technique. The thermal conductivity of the stacked structure at a small Ge fraction was lower than those of the other conventional SiGe materials. Furthermore, in the stacked structure, the thermal resistance per cycle exhibited larger value than that of SK Ge NDs/Si superlattice. The thermal resistance of the stacked structure strongly depended on the Ge ND structure, not on the structure of the Si layer. This indicated that the thermal resistance of the Ge ND interface was the main origin for the reduction in the thermal conductivity, presumably because of the strong phonon scattering at the ND interfaces. This demonstrates that the phonon transport could be controlled by changing the Ge ND structure independently of the structure of the Si layers related to the carrier transport. This study provides a new method to independently control *σ* and *κ*.

## Additional Information

**How to cite this article**: Yamasaka, S. *et al.* Phonon transport control by nanoarchitecture including epitaxial Ge nanodots for Si-based thermoelectric materials. *Sci. Rep.* **5**, 14490; doi: 10.1038/srep14490 (2015).

## Supplementary Material

Supplementary Information
