# Supplementary material for: Phonon transport control by nanoarchitecture including epitaxial Ge nanodots for Si-based thermoelectric materials
Source: Sci Rep. 2015 Oct 5;5:14490. doi: 10.1038/srep14490 (PMC4592960; doi:10.1038/srep14490)
Supplement: Supplementary Information [file srep14490-s1.pdf]

Phonon transport control by nanoarchitecture including epitaxial Ge nanodots for Si-based thermoelectric materials

Shuto Yamasaka, Yoshiaki Nakamura*, Tomohiro Ueda, Shotaro Takeuchi, Akira Sakai

Evaluation of variation of Ge ND structures formed on rough Si layers

To estimate the θ and θ_p in the main text, the structures of the Ge NDs were investigated from scanning tunnelling microscopy (STM) images of Ge NDs in first cycle structure. The use of Ge NDs in first cycle structure for describing the ND structures is based on the similarity of the ND structure in first cycle structure to those in nth cycle structures (n=1-8). The number of NDs evaluated from STM image is large (>~100), which leads to the reduction of statistical error. Therefore, if the situation is that the above-mentioned similarity is confirmed, use of STM image is better for description of ND structures. Here, we confirm the similarity of ND structure using cross-sectional HAADF-STEM (high-angle annular dark-field-scanning transmission microscopy) images.

Figure S1 shows a typical analysis result of the sample of the relatively thin Si layer (~67 ML)/Ge NDs (8 nm) using HAADF-STEM image, which is already reported in our previous paper [J. Electron. Mater. **44**, 2015 (2015).]. This is the case of rough Si layer. The Ge NDs are marked by the red dotted lines. The number of evaluated Ge NDs in one cycle structure is ~10 in TEM images, which is much smaller than the case of STM image. Figure S2 (a)-(c) show the evaluated structure factors of Ge NDs (the height, the lateral size and the projected width). The error is a statistical one which occurred in the evaluation of the structure factors from TEM image. We also checked the variation which arises from definition difference of Ge NDs in images by changing the Ge ND structure definition. As a typical example, one definition of Ge ND structures is shown by light blue dotted line in Fig. S1 and its evaluated structure factors of Ge NDs are displayed in Figs. S2 (d)-(f). Variation due to the definition difference is found to be smaller than statistical errors. These results demonstrated that ND structures in first cycle structure are almost the same as those in nth cycle structures (n=1-8) within the statistical error. In Fig. 2f in main text, the errors of aspect ratio and θ and θ_p include the statistical errors and this structural variation estimated from the above TEM analysis. The projected width should be smaller than the lateral size, but the results in Figs. S2(b) and (c) (also, Figs. S2(e) and (f)) exhibited similar values within the statistical error, which was because the Si layers were not so rough as to make a difference of Ge ND structures.

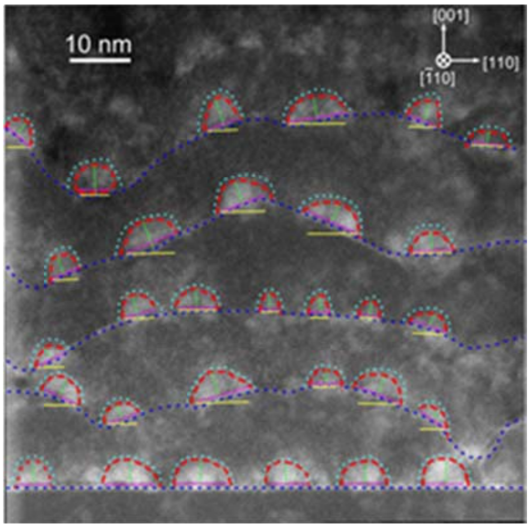

Fig. S1: The analysis of TEM image of the 8-nm/67-ML Si sample. The green, pink and yellow lines are corresponding to the height, the lateral size and the projected width respectively. Red and light blue dotted lines are marks of Ge ND structures, which difference corresponds to the definition difference of Ge NDs structures.

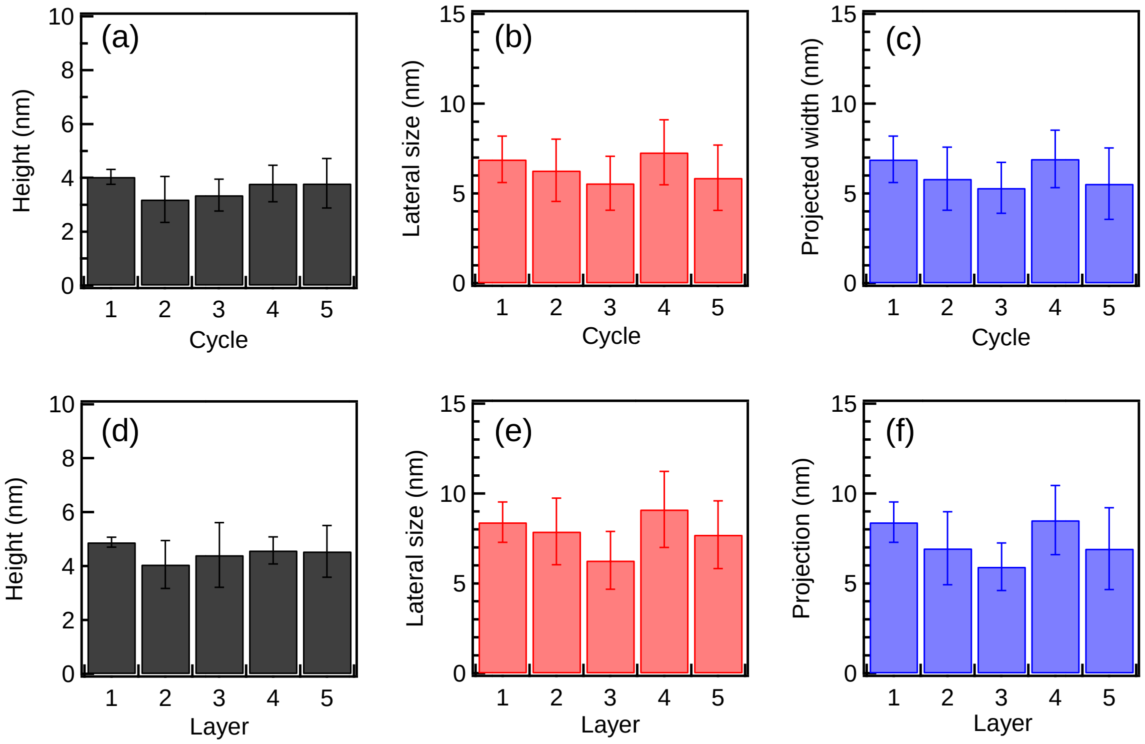

Fig. S2: (a)-(c) The analysis results (height (a), lateral size (b), projected width (c)) of the sample shown in TEM image of Fig. S1 in the case of red dotted marks. (d)-(f) are the analysis results (height (d), lateral size (e), projected width (f)) in the case of light blue dotted marks in Fig. S1.
